# Editorial: Computational and systematic analysis of multi-omics data for drug discovery and development

**DOI:** 10.3389/fmed.2023.1146896

**Published:** 2023-02-21

**Authors:** Shicheng Guo, Dake Zhang, Hu Wang, Qin An, Guangchuang Yu, Junwei Han, Chunjie Jiang, Jianfeng Huang

**Affiliations:** ^1^Department of Medical Genetics, School of Medicine and Public Health, University of Wisconsin-Madison, Madison, WI, United States; ^2^Key Laboratory of Biomechanics and Mechanobiology, Ministry of Education, Beijing Advanced Innovation Center for Biomedical Engineering, School of Engineering Medicine, Beihang University, Beijing, China; ^3^Institute of Cell Engineering, School of Medicine, Johns Hopkins University, Baltimore, MD, United States; ^4^Salk Institute for Biological Studies, La Jolla, CA, United States; ^5^Department of Bioinformatics, School of Basic Medical Sciences, Southern Medical University, Guangzhou, China; ^6^College of Bioinformatics Science and Technology, Harbin Medical University, Harbin, China; ^7^Department of Genomic Medicine, The University of Texas MD Anderson Cancer Center, Houston, TX, United States

**Keywords:** drug discovery, computational biology, bioinformatics, multi-omics, drug development, machine learning, *in silico*

## Introduction

The application of computational analysis in drug discovery is a promising strategy that is accelerating not only the discovery of novel therapeutic targets but also the development of drugs with greater efficacy in disease treatment. Recently, thanks to a tremendous increase in the availability of bioactive macromolecules, advances in understanding of interactions among small molecules, and advances in technologies for computer-aided antibody design, the applicability of computational analysis has been broadly extended to cover nearly every stage in the process of drug discovery and development. This includes identification and validation of the target of interest, lead discovery, assay optimization, preclinical evaluation, and clinical development (1–5). Computational drug discovery methods have evolved significantly over the past decades. Progress in molecular docking, pharmacophore modeling, mapping, *de novo* design, calculation of molecular similarity, and sequence-based virtual screening has enabled quicker, cheaper development of more effective solutions to address fundamental and clinical challenges. Recent advances achieved with the assistance of deep learning methods, as used by AlphaFold (6) and Rosetta (7) in protein structure prediction, constitute another perfect example of how computational solutions can influence drug development strategies. Moreover, computational immunology scientists spent only 6 months developing novel coronavirus vaccines after the first strain was identified. Finally, computational approaches have also been employed to facilitate neoantigen identification (8) and to support personalized cancer immunotherapy in the development of therapeutic cancer vaccines (9–11).

## Overview of articles in this Research Topic

In collating this special issue, we have critically reviewed multiple interesting studies applying computational solutions in diverse translational scenarios, ranging from tumor management to antiviral treatment. These studies span a wide range of research fields, including cancer subtype identification *via* multi-omics, screening of peptidomimetic inhibitors of the NS2B–NS3 protease of the Zika virus, rapid virtual screening of drug compounds from large molecule libraries using the MO-MEMES method, *in silico* screening for SARS-CoV-2 protease inhibitors, *in silico* prediction of cell states and synthetic lethality in cancer using a metabolic network model, and identification of universal tumor markers through pan-cancer analysis.

Lung cancer is by far the leading cause of cancer-related mortality, accounting for almost 25% of all cancer deaths. Tumor heterogeneity, at both the individual and the population levels, is one of the most important findings that has been discovered in recent decades. Identification of disease-specific tumor subtypes has become an urgent priority for precision medicine in the cancer domain. Ruan et al. performed a comprehensive bioinformatics analysis and provide a practical workflow that integrates multi-omics data to identify heterogeneity in lung cancer and cancer subtypes. This study identifies four molecular subtypes, and these findings provide new insight into lung cancer subtypes and potential clinical treatment strategies to guide personalized management. The computational analytic workflow has been deposited in GitHub (https://github.com/ruan2ruan/Multi-omics-Analysis-of-LUAD).

The Zika virus (ZIKV) can cause serious birth defects and brain damage during pregnancy. As a rising public health emergency in recent years, ZIKV infection has attracted international concern. Unfortunately, there is currently neither a cure nor a vaccine for its management. An attractive therapeutic target is NS2B–NS3 protease, which plays an indispensable role in processing the ZIKV polyprotein. Polypeptides can inhibit the functions of target viral proteins, enabling the development of novel antiviral therapeutics (12–15). Using simulations of molecular dynamics, Pant et al. efficiently designed hexapeptides to occupy substrate-binding sites of the protease as novel antiviral candidates against ZIKV.

COVID-19 has become one of the greatest public health challenges; to meet the vast unmet need for COVID-19 medications, Johnson et al. applied a series of computational methods to screen potential small molecular inhibitors from 168 candidate compounds targeting SARS-CoV-2 protease. This strategy combined molecular docking, binding free energy calculations, pharmacophore modeling, induced-fit docking, simulation of molecular dynamics, and ADMET predictions. Additionally, it can easily be applied to other drug targets and larger candidate molecule pools. This study provides a good example of how *in silico* screening could identify potential candidate drugs for further development.

Evaluating the best candidates in a drug library requires comprehensive assessment of multiple aspects of these candidates. Mehta et al. proposed a Multi-Objective MEnhanced MolEcular Screening (MO-MEMES) framework, a machine learning-based framework for identifying the top hits in a drug library based on multiple properties. The framework uses multi-objective Bayesian optimization to identify molecules that have desirable properties on multiple variables, such as synthetic accessibility score, octanol–water partition coefficient, and binding affinity. This molecular docking framework requires only moderate computational resources to efficiently discover more than 90% of the eligible molecules, making it feasible to identify the best candidates even in large drug libraries. With simple adjustments of the parameters and scoring rules, this method can easily be applied to various other problems.

Metabolic differences are among the more important aspects of tumor phenotypes.  Gao et al. integrated metabolic tasks into a multi-objective genome-scale metabolic network model. This model predicted cell states after gene knockout with higher accuracy than a conventional biomass maximization model. The predicted potential single drug targets could potentially be used as biomarkers or become drug design targets. The authors additionally implemented the same multi-objective genome-scale metabolic network model in the prediction of synthetic lethal target pairs of the basal and luminal B subtypes of breast cancer. Through analysis of the predicted synthetic lethal targets, they found that mitochondrial enzymes are potential targets for drug combinations.

Not only can the identification of pan-cancer biomarkers reveal the common molecular mechanisms in tumor development, but such biomarkers also have great potential for use in the clinical diagnosis and treatment of tumors. A comprehensive comparison using indicators widely employed in clinical settings is a direct and inevitable strategy for their evaluation. The set of various indicators relating to tumors is now increasingly complex, including such indicators as immunohistochemical staining results, DNA methylation, gene expression levels, immune response biomarkers, variation in patient survival rates, and so on. Xu et al. propose that the application of machine learning methods may result in better predictive performance in evaluating the roles of chromobox protein homolog 3 in 33 human tumors, while the interpretation of interactions between factors in the model may also pose greater challenges.

## Conclusion

In the near future, it can be expected that the computational and systematic analysis of multi-omics data will continue to play a crucial role in the identification of new therapeutic targets and facilitation of the development of new drugs. With the increasing availability of large amounts of multi-omics data, machine learning and artificial intelligence will likely become more widely used to analyze this data and to identify patterns and relationships that would be difficult to detect through conventional tools and analyses. This will enable a more comprehensive understanding of the underlying biology of diseases and the identification of new drug targets. Additionally, the integration of data from multiple sources, such as genomics, proteomics, transcriptomics, and metabolomics, will provide a more complete and detailed picture of particular disease states and aid in the identification of new drug targets. Furthermore, application of a systems biology approach, in which the interactions between different components of the cell, including proteins, RNA, and metabolites, are studied in a holistic manner, is expected to grow in popularity. This approach aims to understand the complex interactions between different components of the cell and how they contribute to the development of diseases. Such an understanding will help in the identification of new drug targets that may be more effective than traditional targets, and this will also help researchers to understand the underlying mechanisms of resistance to existing drugs. In addition, the use of *in silico* techniques, such as virtual screening, molecular docking, and simulations of molecular dynamics, will continue to play a major role in the identification of new drugs. These techniques allow for rapid screening of large numbers of compounds, reducing the need for expensive and time-consuming *in vitro* and *in vivo* assays.

## Author contributions

All authors listed have made a substantial, direct, and intellectual contribution to the work and approved it for publication.
